# Biological Control of Plant Pathogens: A Global Perspective

**DOI:** 10.3390/microorganisms10030596

**Published:** 2022-03-09

**Authors:** Rachid Lahlali, Said Ezrari, Nabil Radouane, Jihane Kenfaoui, Qassim Esmaeel, Hajar El Hamss, Zineb Belabess, Essaid Ait Barka

**Affiliations:** 1 Phytopathology Unit, Department of Plant Protection, Ecole Nationale d’Agriculture de Meknès, Km10, Rte Haj Kaddour, BP S/40, Menkes 50001, Morocco; said.ezrari@usmba.ac.ma (S.E.); nabil.radouane@usmba.ac.ma (N.R.); jihane.kenfaoui@usmba.ac.ma (J.K.); hajar.elhamss@gmail.com (H.E.H.); 2 Laboratory of Functional Ecology and Environmental Engineering, Sidi Mohamed Ben Abdellah University, P.O. Box 2202, Route d’Imouzzer, Fez 30000, Morocco; 3 Unité de Recherche Résistance Induite et Bio-Protection des Plantes-EA 4707-USC INRAE1488, Université de Reims Champagne-Ardenne, 51100 Reims, France; qassim.esmaeel@univ-reims.fr; 4 Plant Protection Laboratory, Regional Center of Agricultural Research of Oujda, National Institute of Agricultural Research, Avenue Mohamed VI, BP428 Oujda, Oujda 60000, Morocco; zineb.belabess@inra.ma

**Keywords:** biological control, biological control agents, crop diseases, antagonist

## Abstract

The increase in the world population has generated an important need for both quality and quantity agricultural products, which has led to a significant surge in the use of chemical pesticides to fight crop diseases. Consumers, however, have become very concerned in recent years over the side effects of chemical fungicides on human health and the environment. As a result, research into alternative solutions to protect crops has been imposed and attracted wide attention from researchers worldwide. Among these alternatives, biological controls through beneficial microorganisms have gained considerable importance, whilst several biological control agents (BCAs) have been screened, among them *Bacillus*, *Pantoea*, *Streptomyces*, *Trichoderma*, *Clonostachys*, *Pseudomonas*, *Burkholderia*, and certain yeasts. At present, biopesticide products have been developed and marketed either to fight leaf diseases, root diseases, or fruit storage diseases. However, no positive correlation has been observed between the number of screened BCAs and available marketed products. Therefore, this review emphasizes the development of biofungicides products from screening to marketing and the problems that hinder their development. Finally, particular attention was given to the gaps observed in this sector and factors that hamper its development, particularly in terms of efficacy and legislation procedures.

## 1. Introduction

Modern agriculture is continuously developing and evolving. After the generalization of the use of chemical fertilizers and pesticide products, which allowed a considerable increase in yield in the twentieth century, the rise of biotechnologies and new cultivation techniques is underway [1]. The next challenge to be met is to feed around 9 billion people by 2050 [2,3]. In this context, one of the major concerns is to expand food production capacities, including those derived from plants, while preserving the environment [4]. Nowadays, countries are striving to expand their food production to meet their needs [5]. The increase in the production of a given crop is often linked to the improvement of cultivation techniques, particularly the use of more productive cultivars with resistance to main diseases [6].

Crop protection, which is still largely achieved by applying chemical products, is also in a transitional phase [7]. Therefore, the gradual integration of new practices, taking into account the agricultural production system, requires not only the environmental dimension but also the socio-economic dimension [8]. Nevertheless, these cultures are often subjected to parasitic attacks that farmers are still forced to control below the threshold of harmfulness to survive and be efficient [9]. In addition, ongoing growth in productivity and international trade boosts the incidence of certain diseases, thus requiring the application of more pesticides. Subsequently, these pesticides increase environmental pollution and build up chemical residues in the treated ecosystem [10]. Other alternatives such as genetic pathways offer interesting control methods from a practical point of view, but also strengthen the risks of the emergence of resistant genes in the pathogen [11]. Other alternatives such as biological controls using microorganisms are a possible way to minimize the pollution and nuisances associated with the use of synthetic chemicals and greatly reduce their negative impact on the environment [12,13].

The concept of biocontrol has caused an important technological, economic, and political debate aiming to develop sustainable agriculture at a lower ecological cost [14,15]. Accordingly, different countries have implemented a protective plan that can reduce around 50% of used pesticides [16]. These measures unequivocally illustrate a major awareness of the accumulation of toxic residues in the environment and the various links within the food chain. They also indicate the lack of alternatives to reduce the reliance of the agricultural sector on pesticides. In this context, it appears crucial to deepen our knowledge about biocontrols to improve their use and efficiency [15]. For all of these reasons, research is progressing well towards a perspective of biological control, based on the application of microbial inoculum, that could be added to the other aforementioned ways to develop a strong strategy to fight plant diseases.

Promising achievements in terms of biological control have emerged, especially after the successful use of certain antagonistic biocontrol agents (BCAs), in particular *Pseudomonas* spp., *Bacillus* spp., *Burkholderia* spp., and *Trichoderma* sp. against pathogens causing foliar and soilborne diseases like *Agrobacterium radiobacter* var radiobacter, *Erwinia* spp., *Fusarium* spp., *Rhizoctonia solani*, *Phytophthora* spp. and *Pythium* spp. diseases [13,17]. Additional BCAs have showed an antagonistic effect against a wide spectrum of diseases, namely bacterial species such as *Burkholderia* spp., *Paenibacillus* spp., *Pantoea* spp., *Serratia* spp., *Streptomyces* spp., and fungal species such as *Aspergillus* spp., *Beauveria* spp., *Fusarium* spp., *Penicillium* spp., and *Phoma* spp. Furthermore, in addition to halting the pathogen proliferation, many of these BCAs can also directly promote plant growth [9,12,13,17,18]. However, in most cases, the efficacy of BCAs has always been found to be lower than that of synthetic fungicides. This might be due to the complexity of the rhizosphere and the need to apply a high amount of BCAs to cover the entire rhizosphere [19]. This can only happen if the BCA is applied continuously and consecutively. In addition, the way BCAs are formulated and applied can also affect their efficacies [20].

Even though the research for new microorganisms as potential biopesticides has increased, biopesticides have been produced in limited numbers. However, small and medium companies are starting to adapt to this new emerging market. The North American region has the largest amount of the biopesticide market, reaching USD 539 million in 2015 and predicted to attain USD 1.67 billion by 2022 [21]. Research on biological control using microorganisms is experiencing and gaining remarkable momentum, although applications in the field are still limited. The factors limiting the use of BCAs in the field include the inconsistent efficiency in protecting plants under field conditions, decreased availability in the market, and the wide unacceptance of BCAs by farmers.

In this paper, we discuss the progress made on the prospecting of biocontrol agents, their development, and their mechanisms of action, with a special emphasis on legislative procedures and factors affecting their application and marketing development.

## 2. Rhizosphere as a Potential Reservoir of Biopesticides

The rhizosphere is the soil area that surrounds living roots and is influenced by plant root exudates. It is one of the most important microbial hotspots in the soil, with much greater process rates and more intensive interactions [22,23]. The rhizosphere contains a complex microbial community called the rhizosphere microbiome. This microbial consortium lives around plant roots and is formed up of many microorganisms, some of which are beneficial such as bacteria, algae, and fungi [24]. Because it is a source of utilizable carbon, the rhizosphere provides a supportive environment for crucial and intensive interactions between plants, soil, microorganisms, and soil microfauna [25]. Plants can recruit a unique beneficial rhizosphere microorganisms, which can help them to attenuate the disease activity and make them more resistant to environmental stressors [26].

**Figure 1:**
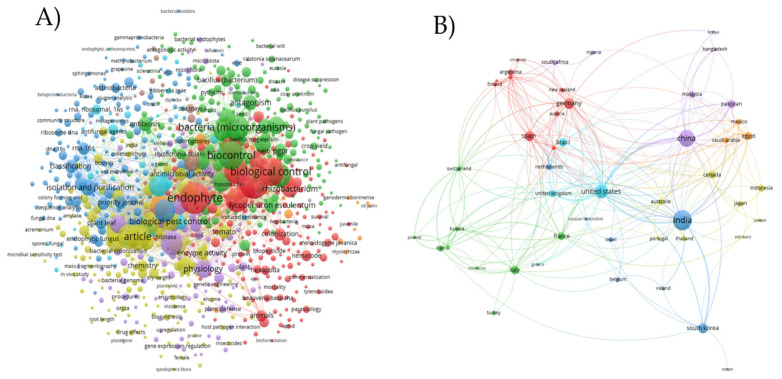
Bibliometric analysis of 1150 articles published on biological control according to the Scopus database using specific keywords such as “Rhizobacteria” OR “Endophytes” OR ”Biocontrol” (**A**) and the network analysis of their worldwide distribution (**B**); the larger the circle, the more intense the scientific activity.

Understanding the role of rhizosphere microorganisms in pest and disease control seems to be a rising research field, as evidenced by the high increase in studies conducted between 2000 and 2019. When the term “rhizosphere” was added to a Google Scholar search with the keywords “microorganisms”, “control”, “pest”, and “diseases”, the number of records retrieved increased from roughly 5000 (2000–2005) to 8500 and >20,000 (2006–2010 and 2011–2019, respectively). In the absence of this term, records dropped from roughly 17,000 to 15,000 in the most recent period [27]. In the present review, we have extracted bibliometric data to perform a deep analysis and establish a network of worldwide distribution-related articles on biocontrol agents. The bibliometric data were extracted from the SCOPUS database (https://www.scopus.com/, accessed on 22 December 2021) using the specific keywords ‘Rhizobacteria’ OR ‘Endophytes’ OR ‘Biocontrol agents’, from which 1150 documents were obtained. The bibliometric analysis was performed using different bibliometric indices, including the most popular used keywords, countries, and the top journals, and was constructed using the VOSviewer processing software (v1.6.9., Leiden University, Leiden, The Netherlands) (Figure 1A). The network analysis showed the worldwide distribution of related articles to biocontrol agents, which revealed the relationship between keywords found and allows the obtainment of a comprehensive perspective of the current research of this area (Figure 1B).

Since the use of pesticides to control pests and diseases has been linked to environmental, ecological, and human health risks, it has become necessary to seek out eco-friendly biological agents known as biopesticides [28,29] to ensure biological control. The latter, often known as “biocontrol”, is a method of reducing or eliminating the impact or damage produced by a specific pest or weed by releasing a biocontrol agent, such as a predator, herbivore, or pathogen [30]. To handle plant diseases, biological control is considered a promising and reliable alternative to the use of synthetic fungicides [27,31]. Several billions of dollars are being spent on biocontrol research, and the number of biocontrol drugs that are available for various plant crops is rapidly expanding [32]. Biopesticides are microbial or products generated from microbes, plants, and other biological organisms aiming to control plant pests [29,33].

Biopesticides have long-term potential in improving sustainable agriculture [29]. There are currently many (more than 440 species) control agents available for various pests [34]. BCAs are used to control plant diseases in crops, and they involve a variety of mechanisms [35]. Van Lenteren et al. [34] presented the first list of BCAs registered worldwide, which includes bacteria, fungi, mycoviruses, and bacteriophages [34]. Certain antagonistic microorganisms have been identified from the rhizosphere of numerous agricultural plants to inhibit some plant diseases and hence reduce the need for agrochemical pesticides.

In the last two decades, researchers have been studying the use of these specialized antagonistic microorganisms in the biological control of soil-borne diseases. Biopesticides are available inside the rhizosphere to combat pests and microbial diseases due to the close connection of root-colonizing probiotic microorganisms with plant host cells [36]. Several bacteria, fungi, protozoa, and nematodes have recently demonstrated antagonistic activity that could be employed in the biocontrol of root and foliar diseases in a variety of crops, and also insect pests [37,38]. Antibiotics, bacteriocins, siderophores, hydrolytic enzymes, and other secondary metabolites produced by these beneficial rhizosphere microorganisms inhibit pathogenic bacteria and fungi [28,32].

Direct antagonism of potential pests through the production of biopesticides is one of the features of plant-probiotic microorganisms that contribute to plant health. Antagonistic fungi such as *Trichoderma* spp., as well as bacteria from the genera *Pseudomonas*, *Bacillus*, and *Streptomyces*, account for the majority of rhizosphere microorganisms commonly used in biocontrols [27].

### 2.1. Beneficial Bacteria

Most plants grown in the field are colonized by various rhizosphere bacteria [39]. Some plant-associated bacteria are classified as beneficial microorganisms, based on their effects on plant performance. Among these free-living bacteria, plant-growth-promoting rhizobacteria (PGPR) thrive freely in the rhizosphere soil [25,33] and produce a variety of antifungal metabolites and plant growth-promoting features [25]. Based on the ability or behavior of the crops and biocontrol agents, PGPRs also act as biopesticides [33]. PGPRs are bacterial species including *Alcaligenes*, *Azospirillum*, *Arthrobacter*, *Acinetobacter*, *Bradyrhizobium*, *Bacillus*, *Burkholderia*, *Enterobacter*, *Erwinia*, *Flavobacterium*, *Pseudomonas*, *Rhizobium*, *Azorhizobium*, *Bradyrhizobium*, *Allorhizobium*, *Sinorhizobium*, *Frankia*, *Mesorhizob*, *Azoarcus*, *Exiguobacterium*, *Methylobacterium*, *Paenibacillus*, and *Pantoea* [29,40,41,42,43,44,45,46,47,48]. In reaction to diverse chemicals found in root exudates, beneficial bacterial communities in the soil are chemoattracted towards plant roots. These beneficial associations boost plant health and crop productivity by increasing nutrient availability, releasing plant growth-stimulating hormones, decreasing pathogen/pest-caused diseases, or improving the environmental stress resistance [39]. Antibiotics, endotoxins, bacteriocins, siderophores, hydrolytic enzymes, hydrogen cyanide (HCN), phenazine-1-carboxylic acid (PCA), 2,4-diacetylphloroglucinol (DAPG), and other secondary metabolites produced by certain rhizosphere bacteria kill pathogens, thus preventing disease development [39,43,44,47,49,50,51,52,53,54]. Some PGPRs utilized in the control of plant pests and diseases are discussed below.

*Bacillus* species have a long history in biocontrol and crop growth-promoting applications [55]. *Bacillus thuringiensis* (Bt) is the most commercially successful biopesticide on the market [29,56]. Bt produces endotoxins [51,52], which are toxic and can be exploited as biopesticides as well as a source of genes for the development of insect-resistant transgenic plants [52]. During their stationary phase of growth, Bt strains produce crystalline proteins (δ-endotoxins) that are toxic to lepidopterous, coleopterous, and dipterous insects as well as mites, nematodes, protozoa, and flukes [51]. Insect bioassays were performed on the spore crystal mixtures of 12 Bt isolates, and it was found that the isolate F8.IIPR has the maximum toxicity against *Spilosoma obliqua* Walker (100%), *Olepa ricini* Fabricius (92%), and *Helicoverpa armigera* Hubner (100%) larvae [57]. *Bacillus subtilis* (BCB-19) and *Bacillus megaterium* (SB-9) induced considerable larval death and growth inhibition in both *H. armigera* and *Spodoptera litura* [58]. *Bacillus amyloliquefaciens* LMR2, *Bacillus halotolerans* (SF3 and SF4), and *Bacillus mojarvensis* SF16 were recently isolated from soil of fire blight host plants in different Moroccan regions for their higher efficacy in reducing apple fire blight disease [48]. *Monilinia fructigena* and *Monilinia laxa*, which cause brown rot disease of fruits, are controlled by *B. amyloliquefaciens* SF14 and *B. amyloliquefaciens* SP10, respectively. In a semi-commercial large-scale study, the efficacy of these strains was found to be comparable to that of two commercial BCAs, but slightly lower than that of a commercial synthetic fungicides [47,59]. The considerable biological control activity of a *Bacillus velezensis* strain named ZW10, as well as its ability to boost host defenses, make it a potential biopesticide for rice blast biocontrol caused by the fungus *Magnaporthe oryzae* [60]. The commercially available *B. amyloliquefaciens* FZB42 product has considerably decreased lettuce bottom rot caused by *R. solani*. This reduction was due to the secretion of surfactin and other FZB42-non-ribosomally synthesized secondary metabolites in the lettuce rhizosphere. Subsequently, plant regulation genes are triggered and expressed to protect plants against the pathogen *R. solani* [53].

In a dual culture bioassay, endophytic *Bacillus* strains obtained from cotton roots have demonstrated an inhibitory efficacy against *Verticillium dahliae* strain VD-080. Scanning electron microscopy examination underlined mycelial disintegration, curling, and shrinkage of *V. dahliae* hyphae after treatment with methanolic extracts of isolated endophytes. Furthermore, when compared to control treatments, cotton plants treated with two *Bacillus* strains (HNH7 and HNH9) showed a considerable reduction in *verticillium* wilt severity. Moreover, the expression of some defense-related genes was significantly higher in plants treated with *Bacillus* strains and inoculated with VD-080 [54]. 

*Pseudomonas chlororaphis* isolates are used as biopesticides in agriculture as they protect plants from various microbial diseases, insects, and nematodes. These isolates directly suppress microbial pathogens, insects, and nematodes by producing a variety of metabolites [36]. *P. chlororaphis* PcO6, isolated from the roots of dryland, enhanced plant health and was used as a biofertilizer and BCA in agriculture. Plant growth is stimulated by an array of metabolites generated by this bacterium through direct pathogen antagonism and induction of systemic resistance in the plant. The mechanisms by which specific bacterial metabolites create protection against pathogenic microorganisms, insects, and nematodes have been identified in studies on PcO6. The role of a global regulatory system, the Gac/Rsm regulon, in conferring protection against plant pathogens has been highlighted [61]. The Gac/Rsm system network in PcO6 communicates the capacities required to adapt to various stressors and enhance survival while also maintaining plant health by priming stress responses. When the bacterium reaches a critical cell density, the Gac/Rsm network is activated, causing the microbe to switch its nutrition consumption and create protectants instead of adding cell mass. Other systems influencing gene expression are linked to the Gac/Rsm system. Antimicrobials are produced when the Gac/Rsm network in PcO6 is activated, which may help in sustaining plant root colonization and protecting the plant against microbial diseases. PcO6 impacts microbiomes and plant health in the rhizosphere by secreting antimicrobials and volatiles. The bacterium forms a protective biofilm on the root surface, which acts as a physical shield and a water-holding gel [61]. Antifungal compounds such as bacteriocin, HCN, and siderophore were produced by *Pseudomonas aeruginosa* isolated from the banana field rhizosphere. Phytopathogens such as *Aspergillus niger*, *Aspergillus flavus*, *Fusarium oxysporum*, and *Alternaria alternata* were suppressed by the isolate’s bacteriocinogenic, siderophoregenic, and HCN rich broth. Bacteriocin has a toxic effect on bacteria, whereas siderophore and HCN inhibit fungal phytopathogens. It is worth mentioning that none of the helpful rhizobia were inhibited by these compounds. In comparison to copper-based systemic chemical fungicide, the *P. aeruginosa* isolate showed higher antifungal activity and a lower minimum inhibitory concentration [43]. The ability of *Pseudomonas* sp. LBUM 223 to control common scab potato caused by *Streptomyces scabies* was proven *via* PCA synthesis. This PCA synthesis is critical for LBUM 223’s capacity to control common scabs of potato, limit pathogen growth, and inhibit the expression of important pathogenicity genes [50]. Similarly, Lanteigne et al. [49] have identified DAPG and HCN as the chemicals responsible for *Pseudomonas* sp. LBUM300’s capacity to inhibit the growth of *Clavibacter michiganensis* subsp. *michiganensis* in vitro and regulate the development of bacterial canker in tomato under soil conditions. In both *H. armigera* and *S. litura*, *Pseudomonas* spp. (SB-21) was reported to induce considerable larval death and growth inhibition [58].

### 2.2. Fungi and Yeasts

Several fungal species have been identified as insect pest entomopathogens, as well as plant endophytic fungi [62]. *Trichoderma* is a ubiquitous fungus genus found as soil inhabitants, plant symbionts, saprotrophs, and mycoparasites [63]. This genus contains filamentous fungi that have been extensively investigated and utilized in agriculture as a biocontrol agent against phytopathogens due to its capacity to compete and parasitize them (mycoparasitism), among other mechanisms of action [62,63,64], as well as mitigating unfavorable growth conditions [63]. *Trichoderma* is by far the most studied fungal biocontrol agent, with some species having previously been commercialized as biopesticides or biofertilizers [63,64]. The use of *Trichoderma* as a BCA for insect pests has been discussed in recent years, both directly and indirectly [62]. *Trichoderma* has been shown to directly suppress insect pests by parasitism and the generation of secondary insecticidal metabolites [62,65], antifeedant chemicals, and repelling metabolites. Indirectly, by activating systemic plant defense systems, attracting natural enemies, and parasitizing insect-symbiotic microorganisms. As a result, *Trichoderma* use in agriculture is effective not only against plant pathogens but also against insect pests, making it a promising future alternative for the development of sustainable agriculture [62]. *Trichoderma aggressivum* isolates were collected from numerous *Agaricus bisporus* growing substrates from farms in Castilla-La Mancha (Spain). This fungus had high antagonistic activity against a variety of phytopathogens, greater than 80%. In a detached leaves assay, the most effective isolate, *T. aggressivum* f. *europaeum* TAET1, has completely suppressed the mycelial growth of fungal pathogens *Botrytis cinerea*, *Sclerotinia sclerotiorum*, and *Mycosphaerella melonis* and inhibited *S. sclerotiorum* sclerotia germination. This excellent compatibility with chemical fungicides may allow this isolate to be used in combination with various pest management strategies [66]. *Trichoderma asperellum* TaspHu1, an isolate obtained from *Juglans mandshurica* Maxim. rhizosphere soils in China improved tomato seedling resistance to *A. alternata* leaf spot disease [67]. *Trichoderma* spp. from olive rhizosphere soils in northern Algeria had great biocontrol potential against *V. dahliae*, the causal agent of wilting on olive trees. These isolates showed an effective potential in reducing the in vitro mycelial growth of this pathogenic fungus. By using the confrontation assay method, *Trichoderma* isolate T12 inhibited *V. dahliae* isolates at a rate of 69% [68]. *Trichoderma* spp. has been proven to be effective in suppressing *Sclerospora graminicola*, the causal agent of pearl millet downy mildew disease. Their prominent role indirectly suppressing the pathogen in the rhizosphere and establishing systemic resistance has been well demonstrated [69].

Furthermore, yeasts are often used in commercial formulations because of their biocontrol capacity. They are already available for postharvest use on a variety of crops, including pome and citrus fruits. Because of their great ability for quick colonization and inhibition of pathogens, as well as their potential for competition for nutrients, physical interaction, and the generation of lytic enzymes, these microorganisms have been shown to have a significant BCA activity [70].

Millan et al. [71] investigated the in vivo and in vitro effects of 69 yeast strains isolated from Spanish vineyards against *Alternaria alternata*, *Penicillium expansum*, and *B. cinerea*, as well as soil-borne diseases *V. dahliae* and *Fusarium oxysporum*, to find a potential yeast with high biocontrol capabilities against postharvest diseases and wilt diseases. As a result, *Wickerhamomyces anomalus* Wa-32 reduced the severity of disease caused by *V. dahliae* up to 40%, and that due to *F. oxysporum* up to 50%. Furthermore, the postharvest assays revealed a high biocontrol performance against *P. expansum* and *B. cinerea* (up to 86 and 97% reduction in disease severity, respectively). Furthermore, *W. anomalus* Wa-32 and two *Metschnikowia pulcherrima* strains showed significant action against *B. cinerea* (Mp-22 and Mp-30). However, according to the same study, *Candida lusitaniae* Cl-28, *Candida oleophila* Co-13, *Debaryomyces hansenii* Dh-67, and *Hypopichia pseudoburtonii* Hp-54 were the most efficient against *P. expansum*, according to the study. This was also observed with grapes and apples [71].

## 3. BCAs Modes of Action

**Figure 2:**
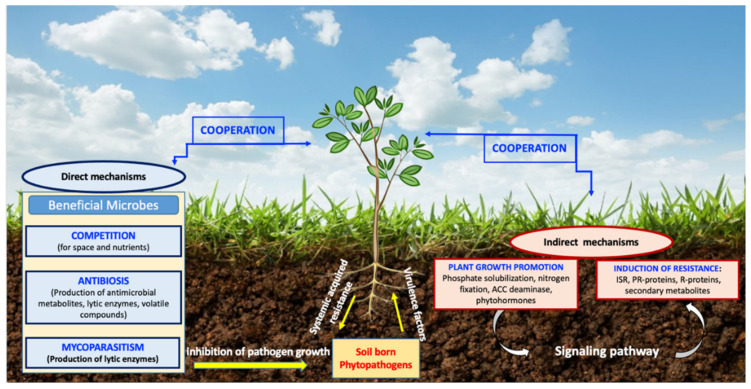
Possible modes of action of biological control agents.

BCAs are applied in the disease management of plant pathogens, where they act *via* a variety of different modes of action to control plant pathogens. Understanding the mechanisms behind the protective effects of BCAs will facilitate the optimization of control and will allow the use of more efficient strains in the correct environment [72,73]. These mechanisms might be used alone or in combination by the BCA to control plant disease directly and indirectly (Figure 2).

### 3.1. Direct Mode of Action

In the direct way of disease control, BCAs act through a direct antagonistic effect on the pathogen, encompassing (i) antibiosis, (ii) parasitism, and (iii) reducing pathogen virulence, and (iv) infection pressure by competition [9,74]. BCAs act through antibiosis that relies on BCAs secretion of allelochemicals which could either be diffusible metabolites such as lipopeptides, bacteriocins, antibiotics, biosurfactants, and cell-wall-degrading enzymes or microbial volatile compounds, which interfere with the metabolism of phytopathogen and thereby inhibit the pathogen development [9,75,76]. There are two types of parasitism: necrotrophic parasitism and biotrophic parasitism. In the first type, the BCA kills the host cells before or just after the invasion of the target pathogen and uses its released nutrients [77]. Whilst in the second type of parasitism, the development of the BCA is favored by a living rather than a dead host structure [78]. Depending on the BCA, we can distinguish the parasitism of viruses on bacteria (bacteriophages), bacteria on fungi (mycophagy), and fungi on fungi (mycoparasitism) [9,75,76]. BCAs may also reduce pathogen virulence by secreting enzymes that interfere with pathogenicity factors, including pectinases and chitinases, which reduce pathogen virulence [76]. Finally, BCAs can reduce pathogen infection pressure through the competition for nutrients and space where both BCAs and pathogens compete with each other. This will help BCAs to establish themselves in the environment through the physical occupation of the site, biofilm formation resulting in reduced colonization of roots by the pathogen, and secretion of the essential micronutrients chelating such as siderophores as well as the characteristics of BCAs, which have more efficient uptake system for micronutrients in the case of the pests [72,79].

#### 3.1.1. Involvement of Lytic Enzymes

Plants are probably primed for SAR by some *Trichoderma* spp., but the full pathway remains inactivated until a subsequent pathogen/parasite attack [80]. That is the case for example of *Trichoderma asperellum* (T203) which was used to prime cucumber against angular leaf spot caused by *Pseudomonas syringae* pv. *Lachrymans*. Disease symptoms decreased by up to 80%, equating to a two-orders-of-magnitude drop in bacterial cell densities in leaves of *T. asperellum*-treated plants. Bacterial cell multiplication in these plants was inhibited. The accumulation of mRNA of two defense genes, the phenylpropanoid pathway gene expressing phenylalanine ammonia-lyase (PAL) and the lipoxygenase pathway gene encoding hydroxyperoxide lyase (HPL), was linked to BCAs protection. This was further corroborated by the accumulation of secondary phenolic compounds, which demonstrated a six-fold increase in the capacity of inhibition of bacterial growth in vitro [81].

*Trichoderma asperellum*, which was isolated from banana wilt-affected plantations, has anti-pathogenic properties based on a variety of mechanisms. One of them includes antibiosis, which is used by a variety of pathogenic fungi, belonging to *Fusarium* genus. *T. asperellum* has been proven to decrease phytopathogen growth by 65–74% and impede spore germination by 30–75%. Furthermore, *T. asperellum* releases mycolytic enzymes (chitinase and β-1,3, glucanase), which may be able to destroy phytopathogen cell walls. In pathogen-induced cultures, the accumulation of respective transcripts and enzymatic activity of both chitinase and β-1,3, glucanase were significantly higher [82]. In the stimulation of the plant immune system by biocontrol fungi against RKNs, ET- and SA-responsive, genes were upregulated by glucanase and chitinase activity, whilst downregulated by genes encoding antioxidant enzymes [80].

#### 3.1.2. Antimicrobial Molecules

Antimicrobial molecules are secondary metabolites that belong to a diverse category of organic, low-molecular-weight compounds produced by microorganisms and are toxic to the growth of other microorganisms or metabolic processes [83]. Antibiotics and related natural compounds can be produced by nearly all living organisms as they are secondary metabolites. They are produced by both prokaryotic and eukaryotic (plant and animal kingdom) species, within unicellular bacteria and eukaryotic fungus. Generally, filamentous actinomyces are the most common and varied producers of antibiotics. Furthermore, amongst prokaryotic and unicellular bacteria, *Bacillus* and *Pseudomonas* species are the most common producers [84].

#### 3.1.3. Biofilm Formation

Understanding how to use BCAs effectively is critically important in sustainable agriculture. The exact mechanisms, chemical or physical, involved when crops are attacked by infectious pathogens are therefore the first steps to determine the factors influencing the efficacity of biocontrol against plant diseases. Physical mechanisms of BCAs, for example, include techniques triggered at the molecular level to physically protect the plant because of antagonistic interactions with host plants. Understanding these mechanisms of action of BCAs would help develop biofungicides products with superior biocontrol properties. BCAs from either the same species or different species can physically interact with surfaces to form complex multicellular assemblies or aggregates of microorganisms also called, biofilms. These biofilms provide various advantages including increased resistance to certain biotic stresses [85]. The microbial cells adhere to each other’s surface through a complex matrix medium comprising a variety of extracellular polymeric substances (EPS) including exopolysaccharides, proteins, and DNA, known as signaling molecules. Microorganisms, including bacteria, cyanobacteria, and fungi, specifically interact with plant tissues through quorum sensing (QS). The latter exists widely in all kinds of microorganisms and is a communication channel for microorganisms [86] and allows individual microbe within colonies to coordinate with each other. In one experiment on *Arabidopsis*, *B. subtilis* 6051 formed a stable, extensive biofilm to protect plants from *Pseudomonas syringae* attacks [87]. Furthermore, both the bacterium *Azotobacter chroococcum* and the fungus *Trichoderma viride* [88] can form biofilms to protect various crops against pathogens [89]. Strains of *B. velezensis* QST713 can also develop a biofilm on inert surfaces to inhibit the growth of *T. aggressivum*, which causes green mold disease [90]. The mechanism of biofilm formation is a powerful tool that helps plants to protect themselves from pathogenic attacks. However, previous knowledge of BCAs and plant species interactions is required before utilization, as BCAs interact specifically with plant species, and thus, the adopted techniques should be species-specific.

#### 3.1.4. Competition for Nutrients and Space

Within BCAs species, competition is defined as the niche overlap of BCAs and pathogens, resulting in simultaneous demand for the same resource [91]. Competition for nutrients (carbohydrates, nitrogen, and oxygen) and space is often suggested as a potential mechanism of action in biological control systems [92]. Fungi, in particular, use the competitive saprophytic ability (CSA), which was originally used to describe the ability of the fungus to colonize dead organic substrates through competition [93]. A fungus with high CSA also exhibits adaptation abilities including the production of antimicrobial substances which are inhibitory to the pathogen growth through both mycoparasitism and the rapid adaptation to environmental triggers (temperature and water availability) [93,94]. Many plant fungal pathogens, including *B. cinerea*, and *S. sclerotiorum*, can be effectively managed through the mechanism of CSA [95]. Both *B. cinerea* and *S. sclerotiorum* are necrotrophic fungal pathogens [96,97]. *B. cinerea* conidia and *S. sclerotiorum* ascospores require external nutrients from plant wounds, senescent plant organs, and/or pollen grains for germination, hyphal development, creation of infection cushions, and infection of plant tissues. Numerous studies highlighted that fungal saprophytes such as *Candida oleophila*, *Clolostachys rosea*, *Epicoccum purpurascens*, *Metschnikowia fructicola*, *Pichia guilliermondii*, *Rhodotorula glutinis*, *Trichoderma harzianum*, and *Ulocladium atrum* are excellent competitors and are therefore effective BCAs of *B. cinerea* and *S. sclerotiorum* [98,99,100,101,102,103,104]. Competition for both nutrients and space is the key physical mechanism used by some BCAs to prevent the development of pathogens.

BCAs decrease the population level of pathogens by depleting food sources as, generally, BCAs are more efficient in nutrient uptake than pathogens [105]. BCAs also use competition for space. Certain microorganisms such as yeasts and bacteria form extracellular polysaccharide capsules that facilitate the adhesion to the fruit surface [106]. Various BCAs limit the amount of nutrients available and reduce the pathogen spore germination percentage [107,108], which subsequently reduces germ-tube growth, infection, and necrosis, and then prevents the expansion of the pathogen [108].

Successful biological controls require colonization by the added biocontrol agents under nutrient deficiency. These BCAs reduced the available nutrients in the wound site and make nutrients inaccessible for the pathogens to germinate, grow, and infect their hosts [109,110]. Iron, for example, is an essential micronutrient that is present in a high percentage of soils. This element, however, has low solubility in soil with a pH > 6, making its acquisition hard for microorganisms [111]. Iron bioavailability has thus become a limiting factor that may lead to competition among organisms. In these conditions, BCAs produce siderophores which are iron-chelating compounds that bind to iron, thus making it inaccessible to pathogens. This iron sequestering mechanism causes inhibition of both growth and metabolic activity of soil-borne pathogens. One siderophore compound in particular, rhodotorulic acid produced by *Rhodotorula glutinis*, improved the control of apple blue rot caused by *P. expansum* [112]. In vitro experiments showed that *Pseudomonas aeruginosa* produced siderophores, in addition to several metabolites with a broad spectrum of antagonistic activities against *Fusarium oxysporium f.* sp. *ciceri*, *F. udum*, and *Aspergillus niger* [113]. Additionally, *Bacillus* species including *B. subtilis* and fluorescent pseudomonads have been effective in rhizosphere colonization [110,114]. *B. subtilis* produced siderophores that played an important role in the control of *F. oxysporum* [115]. In avocado flowers, for example, *B. subtilis* B246 effectively adhered to conidia and hyphae of stem-end rot pathogens and caused cell degradation, thus preventing pathogens from colonizing the flowers [116,117]. This competition mechanism is described in various biocontrol studies with various antagonists such as *Pantoea agglomerans* [118], *Serratia plymuthica* [118], and *Aureobasidium pullulans* [119]. These BCAs were proven to be effective against post-harvest pathogens of fruits [120]. Especially for the control of post-harvest diseases, the use of microorganisms that compete with pathogens for nutrients may be a preferable option to antibiotic-producing microorganisms to avoid food contamination and a build-up of antibiotic resistance within the pathogen population. Generally, for a successful control through competing mechanisms, both the pathogen and the antagonist must have the same requirement for a specific nutrient or resource. The effectiveness of these controls entails that BCAs are present in sufficient quantities at the correct time and location. Overall, the key effects of competition include (i) a reduction of pathogen potential by nutrient competition, (ii) an increase of saprotrophic competition for initial resources in substrate colonization, and (iii) a reduction of the actual amount of the pathogen in either the dormant survival or pathogenic growth phases. Those mechanisms involved in BCA controls can physically protect key crops from devastating pathogens while respecting the environment. Effective use of the correct BCAs is, therefore, an important component of a successful disease management program.

#### 3.1.5. Parasitism

Parasitism is an important biological control mechanism exhibited by antagonists to directly target pathogens that cause soil-borne and foliar diseases [121]. Parasitism or predation occurs when an antagonist feeds on or within the pathogen. It causes the immediate destruction of affected pathogens [122]. Some BCAs use parasitism mechanisms by producing propagules which are aggregates of BCA around or inside the pathogen. The density and the distance between a pathogen and its nearest BCA propagule are important components in effectively controlling pathogens. The denser BCAs are to each other, the more effective they are at controlling pathogens. Many pathogens share the same BCA propagule as their nearest BCA neighbor, and this interaction between BCAs and pathogens helps tremendously in the parasitism process [123]. Some pathogens are resistant to BCAs if optimum environmental conditions are not fulfilled such as adequate temperature and space. BCAs are also influenced by their quality and quantity when introduced to a targeted ecosystem. Pathogen resistance can be determined by measuring the proportion of pathogen propagules that remain infective as a function of the amount of BCAs introduced to the system [124]. Consequently, efforts should be devoted to improving the parasitism process for successful controls. Some of these measures include the use of mixtures of BCAs, optimal timing of antagonist application, integrated biological and chemical controls, and optimization of environmental conditions [124]. The consumption of one fungus by another is called mycoparasitism [125]. Mycoparasitism specifically utilizes fungal cell-wall-degrading enzymes to access the cells [126]. *Trichoderma*, for example, can effectively eliminate phytopathogenic fungi through the suppression of other microorganisms at the same site, making *Trichoderma* the dominant organism in the infested site [127,128,129]. Parasitism between yeasts and fungus was also studied. Cytological damage and protuberances in the cell wall and degeneration of the cytoplasm were observed in vivo culture of both *Candida saitoana* yeast cells with *B. cinerea* mycelium [125]. In 1991, Wisniewski et al. [125] observed a strong in vitro adhesion of the antagonistic yeast *Pichia guilliermondii* cells to *B. cinerea* mycelium [130]. Mechanisms of action determining the success of biocontrol are complex, which, to some extent, may explain the limited effectiveness of biocontrol against plant diseases in field crops. In this case, the screening of these BCAs can be achieved by phenotype-based screenings by evaluating pathogen growth by dual culture assay or by volatile antifungal compounds (VOCs) or by marker-based screening: secretion of antimicrobial metabolites (antibiosis *via* bacterial supernatant), the secretion of cell-wall-degrading or screening bacteria with specific inhibitory mechanisms [76,131].

### 3.2. Indirect Mode of Action

**Figure 3:**
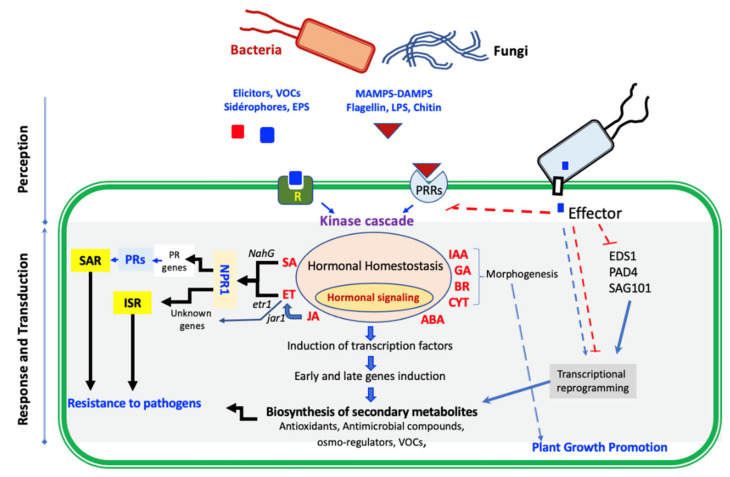
Schematic model of signal transduction events triggered by microbes. Microbes may produce microbe-associated molecular patterns (MAMPs) or damage-associated molecular patterns (DAMPs), such as flagellin or chitin, which are perceived by pattern-recognition receptors (PRRs), or other elicitors, such as volatile organic compounds (VOCs), or siderophores, which are perceived by receptors. The activated receptors may then trigger different signaling cascades, acting as a precursor for the biosynthesis of phytohormones that trigger defensive pathways. The kinase cascade may also phosphorylate transcription factors that modulate the expression of early and late response genes. Abbreviations: 3-indole acetic acid (IAA); abscisic acid (ABA); brassinosteroid (BR); cytokinin (CYT); enhanced disease susceptibility (EDS); ethylene (ET); exopolysaccharides (EPS); gibberellic acid (GA); induced systemic resistance (ISR), jasmonic acid (JA); lipopolysaccharides (LPS); *nonexpressor of pathogenesis-related genes* (*NPR*); pathogenesis-related protein (PR); peptidyl arginine deiminase (PAD), salicylic acid (SA); senescence-associated gene (SAG), systemic acquired resistance (SAR).

Indirect mechanisms include the induction of resistance by stimulating plant defense reactions and stimulating plant growth and soil fertilization [74,132]. BCAs can initiate plant systemic resistance, which results in an accumulation of structural barriers and elicitation of many biochemical and molecular defense responses in the host. This action requires a signalization of the pathway of phytohormones, phytoalexins, and defense enzymes such as phenylalanine ammonia-lyase, chitinase, PR-proteins, and phenolic compounds (Figure 3) [74,133]. Plant defense signaling molecules include salicylic acid involved in defense against biotrophic pathogens and systemic acquired resistance, as well as jasmonic acid and ethylene, both of which are generally considered necessary for defense against necrotrophic pathogens and are beneficial in plant–microbe interactions [134,135]. Inoculation of the plants with specific PGPRs elicits a phenomenon referred to as induced systemic resistance (ISR; Van Loon et al. [136]). ISR provides plants with the ability to withstand attacks by pathogens, which, without bacterial pre-inoculation, would be potentially lethal. Salicylic acid, in response to BCA treatment, activates a hypersensitive response leading to localized programmed cell death that limits pathogen spread, together with the expression of pathogenesis-related genes, which exhibit antimicrobial properties and provide protection against stress [75]. Ethylene is produced when plants are exposed to abiotic or biotic stressors. The 1-aminocyclopropane-1-carboxylate (ACC) synthase is the immediate precursor of ethylene [137]. An increase in the levels of this phytohormone in plants leads to senescence, chlorosis, and abscission [138]. BCAs with ACC deaminase activity can prevent the increase of phytohormone levels in the plant through ACC hydrolysis into α-ketobutyrate and ammonia products, which can be easily assimilated by plants [138]. The application of BCAs may provide tolerance to stress conditions such as (flooding, drought, temperature, and various contaminants), thus allowing plant survival, even under critical conditions [137]. BCAs could also act by stimulating plant growth through the production of plant growth regulators such as gibberellic acid (GA3), indole-3-acetic acid (IAA), cytokinins, and abscisic acid that have a close relation with plant nutrient availability [133]. The contribution to improving plant nutrition could be manifested in a variety of ways, such as the availability of iron (Fe), siderophore production, phosphate solubilization (P), nitrogen (N) fixation, etc. [132].

#### 3.2.1. Induced Resistance and Priming

Plant defense against biotic threats is known to be activated by beneficial microorganisms [80], through a variety of mechanisms, giving plants resistance to diseases [139]. However, the molecular processes through which plants with an activated defense respond to biotic stresses more quickly are still unknown [80]. The defense network of plants is made up of various components that are triggered in response to pathogen attacks. To perform efficiently, this network necessitates a significant commitment at the cellular level, including genetic reprogramming. This includes the stimulation of defense-related genes in plants, such as pathogen resistance genes (PR proteins), as well as the expression of genes encoding specific metabolites or proteins that are involved in the defense setup of the plant system (Figure 3) [140]. For example, the activation of multiple defense responses in the biocontrol activity of several examined BCAs to combat grapevine bunch rot caused by *B. cinerea* and *Aspergillus carbonarius* is indicated by transcriptomic analysis of genes encoding pathogenesis-related proteins PR2, PR3, PR4, and PR5 [141]. Beneficial microbes cause early plant ISR events such as increased expression of pathogenesis-related PR genes, increased activity of defense-related substances such as phenylalanine ammonia-lyase, polyphenol oxidase, peroxidase, β-1,3, glucanase, chitinase, and the accumulation of reactive oxygen species [142,143].

Induced resistance offers the possibility of long-term and broad-spectrum disease control using the natural resistance of plants. The resulting resistance is usually broad and long-lasting but rarely complete, with most inducing agents only lowering the disease prevalence by 20% to 85% [144]. Systemic acquired resistance (SAR) and ISR are two forms of induced resistance that have been characterized based on distinctions in signaling pathways and efficacy spectra [144]. ISR and SAR are two types of plant systemic resistance induced by non-pathogenic and pathogenic microorganisms, respectively [139,145,146]. They frequently overlap with interaction occurring between the two pathways [147]. ISR has emerged as a major method by which bacteria and fungi in the rhizosphere prepare the entire plant body for increased defense against a variety of diseases and insect herbivores [147]. Plants use long-distance systemic signaling to protect distal tissue after ISR activation, inducing quick and powerful immune responses against pathogen invasions [139]. BCAs can therefore suppress pests and diseases by activating the plant immune system [148]. Various BCAs have proven the ability to produce ISR in the past. Beneficial bacteria such as *Bacillus* spp. and *Pseudomonas* spp. can help plants to develop broad-spectrum disease resistance by stimulating defense responses [149,150]. *Bacillus amyloliquefaciens*, *Bacillus atrophaeus*, *Bacillus cereus*, *Pseudomonas fluorescens*, and other bacteria are efficient against fungal, bacterial, and viral invasion by ISR [139]. *Trichoderma* spp. and arbuscular mycorrhizal fungi (AMFs) have long been considered to be widespread potential BCAs [151,152]. A meta-analysis was conducted on papers published between 2010 and 2021 that looked at cross-talk in the tomato–*Trichoderma*–*B. cinerea* system. The analysis was carried out on 15 publications, starting with a collection of 40 papers. *Trichoderma*’s role in the control of grey mold in tomato leaves (decrease in disease intensity, severity, and occurrence, as well as modulation of resistance genes in the host) was highlighted in the research [151]. Defense priming, or AMF-induced resistance, is becoming more widely recognized as AMF’s ability to induce systemic resistance to insect herbivores and diseases [152].

The induction of a distinct physiological state known as “priming” occurs when plants are infected with necrotizing pathogens or when helpful microorganisms colonize the roots of plants. The different cellular defense mechanisms that are triggered during the attack by pathogens or insects, or in reaction to abiotic stress, are activated faster, stronger, or both in primed plants [153]. Plants often switch to a primed state of heightened defense when they detect prospective opponents, invading pathogens, wound signals, or abiotic stress, and some natural or manufactured compounds. The communication appears to vary depending on the considered beneficial microorganism and the elicited plant species [154]. Here, we presented the molecular mechanisms by which some BCAs, especially *Trichoderma*, confer plant protection [63]. *Trichoderma* is a genus of filamentous fungus that colonizes the root surface and play an important role in stimulating plant growth. However, the main proteins and chemical pathways that control this stimulation are still unknown [155]. Several *Trichoderma* spp. can interfere with signaling networks in their host plants to improve disease resistance and stress tolerance, in addition to their ability to directly antagonize plant pathogens and boost plant growth [63]. *Trichoderma* isolates use different strategies to boost the defense pathways of the plant host, depending on their origin and application place. Whilst the phyllosphere *Trichoderma* isolate (BHUF4) used the SAR channel to elicit the defense response in the host plant under *Colletotrichum truncatum* challenge, the rhizospheric *Trichoderma* strain (T16A) used the ISR pathway [140]. *Trichoderma* adapts to different interactions including inter- and cross-kingdom interactions [63]. For example, during the mycoparasitism between *Trichoderma* species and the phytopathogenic fungal infections, several signaling cascades are activated [156]. Important processes in *Trichoderma* mycoparasitism are adapted such as the development of infection structures called appressoria during mycoparasitism, the generation of hydrolytic enzymes, antimicrobial metabolites, and the induction of systemic resistance in plants. All these processes rely on signaling pathways that are activated by the binding of host-derived ligands to receptors [157].

#### 3.2.2. Implication of Phytohormones

Several low-molecular-weight compounds known as phytohormones, such as SA, JA, and ET, influence the immune response in plants. To trigger the plant immune response against pathogen and parasite attacks, BCAs use a variety of sophisticated molecular processes [80]. SAR is one of the most well-studied mechanisms, which provides long-term protection against a wide range of microorganisms [158]. SAR confers long-term resistance to (hemi) biotrophic pathogens and pests and is mediated by SA. It is linked to the activation of pathogenesis-related (PR-) genes. Rhizobacteria ISR is largely effective against necrotrophic diseases and herbivorous insects and is regulated by JA and ET. It is not related to changes in PR-gene expression [147,159]. The transmission of ISR signaling was formerly thought to be dependent on JA and ET, but not on SA. The involvement of both the SA and JA/ET signaling pathways, as well as the regulatory roles of small RNA in ISR, has been revised in the previous decade [139]. *T. harzianum* T22 improves plant direct resistance against stink bug (*Nezara viridula*) feeding attack by increasing JA marker gene transcript levels early. Tomato plants were shown to respond to *N. viridula* at the molecular level. *N. viridula* feeding activates the JA defensive signaling pathway, as evidenced by an increase in ToLOX D expression after 8 h of stink bug feeding. ToPIN2 was also highly elevated 8 h after herbivore feeding, most likely because of the JA-cascade being activated. Upregulation of ToPIN2 may play a role in the lowered growth rate of stink bug nymphs [160]. Beneficial root endophytes, such as *Trichoderma* and *Glomus* spp., have been proven in numerous studies to minimize endoparasitic nematode infections by eliciting the plant immune system [80,161,162]. When attacked by root-knot nematodes (RKNs), tomato plants treated with *T. harzianum* had their SA-signaling pathway and ET biosynthesis activated, which helped in controlling the infection. Monitoring the expression of the genes PR-1/PR-5 and JERF3/ACO, which are indicators of the SA- and JA/ET-dependent signaling pathways, respectively, revealed this effect. Five days after nematode inoculation, roots of plants pre-treated with *T. harzianum*-strains showed an over-expression of PR-1, PR-5, and ACO genes. In *T. harzianum*-colonized plants challenged with nematodes, JERF3 gene expression remained unchanged [161]. Plants are primed against RKNs through BCA contact with roots. BCA-mediated immunity appears to be dependent on SA-mediated SAR and is linked to both the activation and inhibition of chitinase and glucanase enzyme activities, as well as the inhibition of the plant antioxidant enzyme system [80].

Hence, the screening of such mechanisms can be based on phenotype-based screenings by measuring plant growth and root colonization traits, a reduction in disease severity, and alleviating abiotic stress. It can also be based on marker-based screening, either by using specific medium culture for BCAs screening or by searching the presence of genes responsible for these mechanisms [76]. Some other techniques require a high-throughput tool, such as induced systemic response markers, expression of pathogenesis-related) proteins at the transcriptomic level, and the production of reactive oxygen species (ROS) [76,79]. BCAs with multiple modes of action are highly demanded. The nature of the mode of action of selected BCA is among the data requirements for the registration of BCA-based products.

## 4. Biological Control: Facing Reality

The modern application of biological control was introduced at the end of the nineteenth century; however, related techniques were in use for at least 2000 years [34]. Biological control is composed of four different types, including conservation, natural, classical, and augmentative controls [163]. The method is widely considered an attractive, eco-friendly alternative for pest management [164].

Although the strategy offers a promising alternative to synthetic pesticides in the control of pests and plant disease, the method faces many challenges. Serious ecological consequences such as outbreaks could be associated with the introduction of non-native living species that could become invasive and cause significant deleterious impacts on the environment [164]. Moreover, the use of BCAs has not always been successful, probably due to changes in environmental conditions. *Trichoderma* sp., for example, showed its predatory behavior only under limited nutrient conditions. It has been reported that the *Trichoderma* spp. do not attack *R. solani* in the presence of compost, which gives the availability of cellulose as nutrients for the agent [164].

Even if the evidence about the effectiveness of biopesticide is reported to be satisfactory, the availability of such products is still not well established in the market. Additionally, the commercialization of plant-based biopesticide highly depends on the availability of plant sources in large quantities and cultivation. Moreover, a plant could have many active substances, which makes biopesticide formulation a very challenging task [165]. Several other issues are related to the use of BCAs, including the shelf life of biological pesticides which are known to have a high rate of biodegradability. Furthermore, pesticide-based microbes may not be efficient on all pests in the field; they only control a small portion of pests [165].

### 4.1. Biopesticide from Lab to the Field

The screening of potential strains is part of specialized laboratories’ mission to develop biopesticide products that are highly efficient against plant pathogens in agriculture [166]. These strains are generally selected based on their effectiveness against pathogens, host range, availability, ease for mass production, formulation, and application by farmers. The strains that had the lowest LC50 and LT50 are tested under greenhouse and later in controlled field experiments. The formulation and field efficacy trials are conducted by partners from the private sector and accredited laboratories, who conduct the required eco and mammalian toxicity tests and quality assurance for later commercialization of biopesticide [167]. However, field bioassays are required to confirm the efficacy of selected products.

The biopesticides are classified by governments so that the authorities can regulate their use through authorizations. The main focus of this regulation is the environment, human safety, and the reliability of the product. The efficacy and labeling of the biopesticide also should meet the requirement set up by the authorities and EU for their safe handling [168]. The regulation portfolio of biopesticide registration is normally a modified version of the conventional chemical pesticides, with risk assessment. That includes toxicological and ecotoxicological evaluations, their mode of action, and host range. These requirements can be challenging for regulators. One of the challenges is identifying the appropriate biopesticides while at the same time ensuring safety and consistency standards that are acceptable for commercialization [168].

### 4.2. Limited Number of Registered Products

An increase in agricultural productivity has occurred in the latest decades due to population expansion and, as a result, the demand for food [169]. To date, the consumption of chemical fertilizers and pesticides has led to ecological imbalance and the contamination of natural resources [170]. Sustainable agriculture is the solution to problems arising from decades of uncontrolled use of chemical-based agronomic techniques to boost crop yields. As a result, an eco-friendly substitute for chemical fertilizers and pesticides is a necessity. With their diverse and unique capabilities, microbial agents represent an appealing and realistic option to substitute chemicals [171].

BCAs influence the interactions between plants, pathogens, and their environments, resulting in biological and physical pathways that affect pathogen fitness, plant health, and ecological function. These interconnections create a panorama of compromises between natural and social functions of biological control, therefore requiring a full assessment of its benefits and costs from societal and farmer perspectives for their long-term development and deployment [15]. BCAs have shown significant results against a wide range of phytopathogenic fungi, oomycetes, bacteria, and weeds. They constitute a promising alternative to synthetic pesticides, although their application in agriculture remains remarkably scarce.

**Figure 4:**
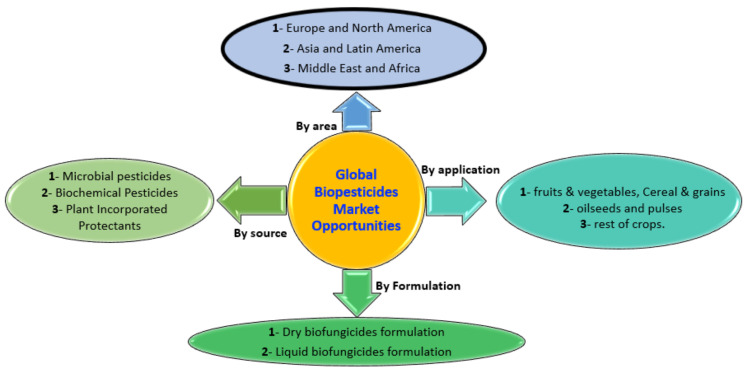
The potential market of biopesticide.

Commercially accessible biocontrol solutions that control plant disease, unlike insect biocontrol, are a novel potential. The first bacterium, *A. radiobacter* strain K 84, was enlisted by the United States Environmental Protection Agency (EPA) in 1979 to combat crown gall disease in plants. A decade later, the EPA approved the first fungus, *T. harzianum* ATCC 20476, to manage plant diseases. Currently, 14 bacteria and 12 fungi strains have been recorded by EPA that aid in treating plant disease [172]. The plurality of these BCAs was commercialized as one or more products on the market. Commercialization technology is still in its beginning phases (Figure 4). Most of the EPA-registered species (64%) were recorded in the last decade, while the remaining 36% were documented within the last five years. For these products to reach the market, several technological challenges were surmounted. Table 1 lists some commercially available biocontrol products on the market [173,174].

To date, BCAs have a commercial value of less than 5% of the whole crop protection industry [175], despite their well-known documentation. The limited number of licensed products for biocontrol of plant diseases is significantly linked to the low technology transfer, implying that the agricultural sector, mainly in developing countries, has yet to recognize its economic potential. Besides that, for promising microbial agent candidates, sufficient knowledge of organism-specific research methods for mass manufacturing and conceptualization is often lacking, making mass production of the entire microbe in in vivo conditions an expensive and time-consuming task. The cost of this approach is a major constraint, as its production and licensing are too expensive when compared to chemical agents [174,176]. Furthermore, ensuring BCAs reach right area at the right time and with sufficient density to be effective, as well as keeping them permanently there, represent one of the most challenging aspects of their use. Since biocontrol involves the introduction of non-native living organisms, serious ecological impacts may be associated with them. Non-native species, for instance, may become invasive and have harmful environmental consequences if they spread beyond the area where they were introduced.

Moreover, some BCAs exhibit efficacy under in vivo circumstances in the laboratory, but ecological restrictions hamper their performance under real full-scale conditions [177,178,179], making them economically non-viable with chemical pesticides. Given the increasing number of microorganisms used for biological control, few are recorded against plant diseases in the EU, according to the European project ENDURE (European Network for Durable Exploitation of Crop Protection Strategies). Several studies found that when certain BCAs were introduced under commercial field circumstances, they were less effective or altogether ineffective, despite their high performance in controlled conditions [45,180,181,182]. For example, many *Pseudomonas* BCAs showed a good performance in trials but cannot be translated into consistent, efficient disease control in various field settings [182]. For the antagonist, *Candida oleophila* strains, a significant difference in enzymatic activity existed between in vivo and in vitro applications [15,183]. 

Despite the fact that BCAs are genetically stable, their use has not been very successful due to ongoing climate change. As a result, it is critical to choose agents that are effective across a variety of environmental situations (soil texture, temperature, humidity, radiation). A small change in temperature amends the microbial concentration. The virulence of the agents is decreased when they are exposed to UV rays from the sun. Some BCAs only exhibit predatory behavior when nutrients are scarce rather than in normal growth conditions. For example, *Trichoderma* spp. does not directly attack the *R. solani* when fresh bark compost is introduced. The availability of cellulose itself is a reason for this. When lower amounts of cellulose, genes governing chitinase synthesis, in *Trichoderma* spp. are activated, the enzyme for parasitic activity is then produced [164,184].

Since BCAs have a limited shelf life, their viability must be managed. For instance, *Trichoderma viride* is viable for four months, while *Pseudomonas fluorescens* can only survive for three months [164,185]. *Bacillus* is thought to be a microbiological factory that generates a large set of antimicrobial substances and is found in about 85% of commercially available BCAs [175,186]. Although basic fermentation and formulation processes can create this type of formula, the commercial applicability of such procedures is limited. This is due to a lack of acceptable methods for large-scale manufacturing and appropriate technology to address the problem of short product shelf life. Maintaining a high spore density is important to achieve optimal sporulation efficiency, which is a viable solution to this problem. As a result, the entire production process must be streamlined in generating an efficient and high-quality product [187,188].

The evolvability of target pathogens is negatively correlated with the endurance of BCAs [189]. BCAs targeting pathogens with higher genetic variation from sexual reproduction, large effective population size, and long dispersal ability are expected to have a shorter durability [190]. Continuous commercial use of the same BCAs might lead to a strong selection of infections, resulting in the emergence of new pathogen populations that can evade or attenuate the negative effects of the BCAs [15,45]. Moreover, because it is difficult to estimate the specificity of the age and stage of targeted pathogens, the time of application of BCAs must be established [174,185].

Another aspect limiting the amount of BCAs registered is growing skepticism regarding their efficacy. Many farmers, particularly in underdeveloped countries, are hesitant to adopt the practice due to technical difficulties, environmental concerns, or economic appeal [45,75,191]. They choose which plant disease management technologies to implement, and their viewpoints on biological control are influenced by economic, technological, and practical considerations. Examples of the latter include effectiveness, profit, availability, and convenience. To entice farmers to use biological control, technology must be simple to assess and prepare, whilst providing economic advantages over alternative options in terms of cost, demand, and supply efficiency.

**Table 1:** Biopesticides, their trade names and targets [18,172,173,174,182,192,193,194,195,196,197].

Biopesticides Active Agents	Trade Name	Target Disease and/or Target Organism Pathogen	Crop	Manufacturer/Distributor
A. Bacteria biopesticides				
*Agrobacterium radiobacter* strain k84	Galltrol	*Agrobacterium tumeifaciens*	Ornamentals, Fruits, Nuts	AgBioChem, Los Molinos, CA, USA
*Bacillus subtilis* QST 713	Serenade	Foliar pathogens, rots, Fire blight, and blights	Cherries, cucurbits, grapes, leafy vegetables, peppers, potatoes, tomatoes, and walnuts	AgraQuest, Davis, CA, USA
*Bacillus firmus* NCIM 2637	Bionemagon	Root-knot nematode, Remiform nematode Cyst nematode, Burrowing nematode, Lesion nematode	Cereals, millets, pulses, oilseeds, fibre crops, sugar crops, forage crops, plantation crops, vegetables, fruits, etc.	----
*Bacillus subtilis* GB03	Companion, Kodiak	*Fusarium*, *Pythium*, *Rhizoctonia*, *Aspergillus*, and others	Crop seeds, including seeds for cotton, peanuts, soybeans, wheat, barley, peas, and beans	Growth products, White Plains, NY, USA
*Bacillus subtilis* MBI 600	Subtilex; Histick N/T	Damping-off *Fusarium*, *Rhizoctonia*, *Alternaria*, and *Aspergillus*	Cotton, beans, barley, wheat, corn, peas, peanuts, and soybeans	Becker Underwood, Ames, Iowa, USA; Premier Horticulture, Quakertown, PA, USA
*Bacillus subtilis* var. *amyloliquefaciens* strain FZB24	Taegro	*Rhizoctonia* and *Fusarium*	Shade and forest tree seedlings, ornamentals, and shrubs	Earth BioSciences, Salem, OR, USA
*Bacillus licheniformis* strain SB3086	Ecoguard; Novozymes Biofungicide GreenRelief	Foliar pathogens and blights	Ornamental plants and ornamental turf	Novozymes Biologicals, Davis, CA, USA
*Bacillus pumilus* strain GB34	GB34 Concentrate Biological Fungicide	*Rhizoctonia*, *Fusarium*	Soybean	Gustafson, Inc, Plano, TX, USA
*Bacillus thuringiensis*	Bio-Dart, Biolep, Halt, Taciobio-Btk, Tacibio, Technar	Lepidopteran pests	Stored grains, fiber, and food crops	----
*Bacillus thuringiensis tenebrionis*	Novodor, Trident	Colorado potato beetle	Potato	----
Burkholderia spp. strain A396	Venerate	Aphids, leafhopper, lygus, stink bug, thrips	Almonds, blueberry, citrus crops, cucurbits, fruiting, vegetables, grapes	----
*Chromobacterium subtsugae*	Grandevo WDG	Armyworms, Aphids, Asian Citrus Psyllid, Mites, Spotted Wing Drosophila, Thrips, Whiteflies.	Blueberry, citrus crops, cucurbits, fruiting vegetables, grapes, leafy greens	----
*Pseudomonas chlororaphis* strain 63–28	AtEze	*Pythium*, *Rhizoctonia solani*, *Fusarium oxysporum*	Vegetables and ornamentals in greenhouses	EcoSoil Systems, San Diego, CA, USA
*Psuedomonas fluoroscens* strain A506	Frostban	Fire blight, bunch rot	Fruit crop, tomato, potato	Plant Health Technologies, Burlington, CO, USA
*Pseudomonas aureofaciens* strain TX-1	Bio–Ject, Spot Less	*Rhizoctonia solani*, *Sclerotinia homeocarpa*, *Colletotrichum graminicola*, *Pythium aphanadermatum*, *Michrodochium nivale*	Vegetables and ornamentals in greenhouses, golf course turf	EcoSoil system, Canyon Lake, TX, USA
*Pseudomonas fluorescens* A506	BlighBan A506; Frostban	Frost damage, fire blight, bunch rot	Fruit crops, almond, potato, and tomato crops	Frost Technology Corporation, St Croix Falls, WI, USA; Plant Health Technologies, Burlington, CO, USA
B. Fungi and Yeast Biopesticides				
*Ampelomyces quisqualis* isolate M-10	AQ10 BioFungicide	Powdery mildew	Fruit, vegetable, and ornamental crops	Ecogen, Grand Junction, CO, USA
*Aspergillus flavus* AF36	Aspergillus flavus AF36	*Aspergillus flavus*	Cotton	Arizona Cotton Research and Protection Council, Phoenix AZ, USA
*Aspergillus flavus NRRL21*, *882*	Afla-guard	*Aspergillus flavus*	Peanut	Circle One Global, Cuthbert, GA, USA
*Beauveria* spp.	Biosoft, ATEC Beauveria, Larvo-Guard, Biorin, Biolarvex, Biogrubex, Biowonder, Veera, Phalada 101B, Bioguard, Bio-power, Myco-Jaal	Coffeeberry borer, diamondback moth, thrips, grasshoppers, whiteflies, aphids, codling moth	Coffee berries, canola, mustard, cruciferous vegetables, and others	----
*Chaetomium globosum*	Ketomium	Rice blast, durian, and black Pepper rot, citrus rot, strawberry rot, anthracnose, and others	Rice, black pepper, citrus, strawberry, tomato, corn, and others	----
*Coniothyrium minitans* CON/M/91-08	Contans WG; Intercept	*Sclerotinia sclerotiorum* and *Sclerotinia minor*	Agricultural soil	PROPHYTA Biologischer Pflanzenschutz GmbH, Wismar, Germany; Technology Sciences Group, Sacramento, CA, USA
*Gliocladium catenulatum* strain JI446	Prima stop soil guard	Soil-borne pathogens	Vegetables, herbs, spices, turf, ornamentals, tree, and shrub seedlings	Kemira Agro Oy, Helsinki, Finland; RegWest Co., Holland, MI, USA
*Gliocladium virens* GL-21	SoilGard	Soil-borne pathogens	Ornamentals, vegetables, cotton	Thermo Trilogy Corporation, WALTHAM, MA, USA
*Gliocladium virens*	SoilGard 12G	Clubroot *Plasmodiophora brassicae*	Canola and crucifer vegetable crops	Certis USA L.L.C., Columbia, MD, USA
*Metarhizium anisopliae*	Meta-Guard, Biomet, Biomagic, Meta, Biomet, Sun Agro Meta, BioMagic, ABTEC, Verticillium	Coleoptera and Lepidoptera, termites, mosquitoes, leafhoppers, beetles, grubs	Cotton, vegetable, field crops, and others	----
*Pseudozyma flocculosa* strain PF-A22UL	Sporodex L.	Powdery mildew	Roses and cucumbers in greenhouses	Plant Products Co., Leamington, ON, Canada; Technology Sciences Group, Washington DC, USA
*Streptomyces lydicus*	Actinovate AG	Clubroot *Plasmodiophora brassicae*	Canola and crucifer vegetable crops	Natural Industries Inc., Houston, TX, USA
*Streptomyces griseoviridis* strainK61	Mycostop	Soil-borne pathogens	Ornamentals, tree seedlings	Kemira Oy, Helsinki, Finland
*Trichoderma harzianum* ATCC 20, 476	Binab T	Tree wound pathogens	Wounds in ornamental, shade, and forest trees	BINAB Bio-Innovation AB, Helsingborg, Sweden
*Trichoderma album* 2.5%	Bio Zeid	Tomato wilt *Fusarium oxysporum*	Tomato	----
*Trichoderma harzianum* T39	Trichodex	*Botrytis cinerea*	Most of the food crops	Bio works, Victor, NY, USA
*Trichoderma harzianum* T22	Root shield, plant shield	Soil-borne pathogens	Greenhouse nurseries	Bio works, Victor, NY, USA
*Verticillium lecanii*	Verisoft, ABTEC, Verticillium, Vert-Guard, Bioline, Biosappex, Versitile, Ecocil, Phalada 107 V, Biovert Rich, ROM Verlac, ROM Gurbkill, Sun Agro Verti, Bio-Catch	Whitefly, coffee green bug, homopteran pests	Coffee crops and others	----
C. Postharvest biofungicides				
*Candida oleophila*	Aspire	*Botrytis*, *Penicillium*, *Monilinia*	Pome fruit, citrus, strawberry, stone fruit	Ecogen, Grand Junction, CO, USA
*Candida oleophila* isolate I-182	Aspire	Postharvest diseases	Various fruits, vegetables, flowers, ornamentals, other plants	Ecogen, Grand Junction, CO, USA
*Cryptococcus albidus*	YieldPlus	*Botrytis*, *Penicillium*, *Mucor*	Pome fruit, citrus	Lallemand, Bellville, South Africa
*Candida sake*	Candifruit	*Penicillium*, *Botrytis*, *Rhizopus*	Pome fruit	IRTA/Sipcam-Inagra, Valencia Spain
*Pseudomonas syringae*	Biosave	*Penicillium*, *Botrytis*, *Mucor*	Pome fruit, citrus, strawberry, cherry, potato	Jet Harvest Solutions, Longwood, FL, USA
*Bacillus subtilis* *Candida oleophila*	Avogreen Nexy	*Cercospora*, *Colletotrichum* *Botrytis*, *Penicillium*	Avocado Pome fruit	South Africa Lesaffre Belgium
*Aureobasisium pullulans*	BoniProtect	*Botrytis*, *Penicillium*, *Monilinia*	Pome fruit	Bio-ferm, Herzogenburg, Austria
*Pantoea agglomerans*	Pantovital	*Botrytis*, *Penicillium*, *Monilinia*	Citrus, pome fruit	IRTA/Sipcam-Inagra, Valencia Spain
*Metschnikowia fructicola*	Shemer	*Botrytis*, *Penicillium*, *Rhizopus*, *Aspergillus*	Table grape, pome fruit, strawberry, stone fruit, sweet potato	Bayer/Koppert, The Netherlands

Some farmers prefer formulations in granules that can be stored and easily applied by machines over those that require more expensive equipment or cold storage. Farmers are discouraged from using the biological control strategy due to a lack of awareness of technological features and a limited number of successful technologies. In this scenario, training and field demonstrations are crucial for exchanging information between technology developers and end-users. Biological management has a direct impact on farmers’ expenses and revenue, but it also has an indirect impact on their economic benefits through its effect on the ecological function and sustainability of farmland [15]. On the other side, it became evident that current regulation and registration procedures for microorganisms, which are mostly based on those for chemical pesticides, are not well suited. This has contributed to the slow implementation of BCAs [176]. To summarize, the full potential of BCAs was not realized, mostly because research is only confined to the laboratory, and thus, little emphasis has been dedicated to developing commercial formulations of BCAs. Furthermore, because of a lack of information on how to use what has been commercially produced, farmers have not used it effectively. As a result, there is a need to strengthen the notion of biological control extension to popularize the concept.

### 4.3. Legislative Procedures

Although the number of biocontrol products used in plant disease management is growing, they still account for just 1% of agricultural control measures, while synthetic fungicides account for 15% of total chemicals used in agriculture [172].

The commercialization of biofungicides is a multi-step process with several restrictions. BCAs, like synthetic pesticides, are submitted to risk assessments before being approved for commercialization. The European Regulation (EC) No 1107/2009 specifies rules for the marketing of plant protection products based on a risk assessment. Regulation (EC) No 540/2011 establishes a list of authorized microorganisms for biocontrol usage in Europe. However, current risk assessment approaches are not entirely adapted to the extremely difficult task of assessing the safety and dangers of such living substances. Eventually, instructions on how to conduct BCA risk assessments are required [198].

Regulation EC1107/2009 took effect in June 2011 to prevent the approval of substances that present unacceptable risks to human/animal health and the environment. As a result of the revised registration standards, some active compounds will not be reapproved due to their classification (cut-off criteria). When a substance meets one of the following environmental requirements, it cannot be registered: persistent organic pollutant (POP); persistent, bioaccumulative, and toxic (PBT); and/or highly persistent, very bioaccumulative (vPvB). These toxicological criteria will also be used to eliminate substances classified as carcinogens (C1A and C1B), mutagens (M1A and M1B), or reproductive toxins (R1A and R1B) [199].

Regulation EC 1107/2009 (2011)-Article 4 (3) claims that a plant protection product shall meet the following requirements: (a) it shall be sufficiently effective; (b) it shall have no immediate or delayed harmful effect on human health or animal health, directly or through drinking water, food, feed or air, or consequences in the workplace or through other indirect effects or on groundwater; (c) it shall not have any unacceptable effects on plants or plant products; (d) it shall not cause unnecessary suffering and pain to vertebrates; and (e) it shall have no unacceptable effects on the environment, having particular regard to the following considerations: (i) its fate and distribution in the environment; (ii) its impact on non-target species; (iii) its impact on biodiversity and the ecosystem [199].

Finally, commercial authorizations for biopesticides (and synthetic pesticides) are awarded after a laborious process [9,199] requiring a set of tests, such as toxicological and environmental investigations, as well as efficiency. In many circumstances, toxicological studies do not exist or are too expensive and time-consuming for local manufacturers [168,197]. Nonetheless, the present trend toward lessening the usage of synthetic pesticides and easing the regulatory process for low-risk compounds may allow BCAs to be produced and utilized globally [200].

## 5. Factors Affecting the Success/Failure of Biological Control of Plant Pathogens

Despite decades of research, the importance of biological control in plant health management has remained relatively minor in the face of rising demand for alternatives to chemical control. In laboratory conditions (in vitro or *planta*), reports of a substantial effect were significantly more common than in field trials. In comparison to chemicals, it is widely understood that moving from the controlled conditions of a laboratory experiment to the harsh conditions encountered in the field has proven more difficult for biopesticides. Whilst the BCA field efficacy can match or exceed that of chemical pesticides, this can vary over time and from one area to another [201]. In other words, the antagonist that controls or suppresses pathogens in the laboratory is rarely effective in the field. This is due to the antagonist, the pathogen, the host, the environment, and all the intricate interactions that occur between them. The host, for example, passes through a series of evolutions or mutations, changing its physical, biological, and chemical features. The antagonist’s efficacy is also determined by pathogenic features. The antagonist may change in response to changes in environmental conditions, population, and the presence of microbial colonizers in the biological system [32]. For example, although *Trichoderma* is by far the most widely studied fungal BCA, its widespread use has been impeded by its unpredictability in the field. Understanding how *Trichoderma* interacts with plants, other microorganisms, and the environment is critical for developing and implementing *Trichoderma*-based agricultural production and protection strategies [63].

### 5.1. Effect of the Plant on Biocontrol Activity

The outcome of biological control can be heavily influenced by plant species and genotypes [202]. Umer et al. [32] widely documented the plant effect on biocontrol activity. The plant itself serves a dual purpose in biocontrol [32]. The degree of rhizosphere colonization and antibiotic production by antagonists, as well as the development of induced resistance by plants, can all be influenced by plant genotype. The plant susceptibility to some nematode species, for example, can influence the effectiveness of control; good hosts will require more suppression than bad hosts [202]. The plant also serves as a place of action for pathogens and antagonists, where they interact. Pathogen and antagonist interactions are influenced by the host exudate excretion, ion, and water absorption, gaseous exchange, and surface temperature [32]. When plant genes are expressed, they have an impact on the microbial community on the surface of the plant and its surrounding space.

### 5.2. Effect of the Pathogen on Biocontrol Activity

Pathogen behavior is one of the most significant factors to consider when choosing BCAs; due to genetic variability and ecological fitness diversity, each pathogen has a unique behavior interaction with the host. It is important to highlight that pathogen behavior differs from that of the antagonist due to both its pathogenicity and susceptibility to antagonist action [32]. The persistence of a plant protection control efficacy in space and time is referred to as its durability. It depends on two factors: (i) the selection pressure exerted on plant-pathogen populations and (ii) the pathogen’s ability to adapt to the control strategy. In other words, regardless of the complexity of their mode of action, plant pathogens can show a wide range of sensitivity to BCAs (including very low sensitivity). Certain pathogens can adapt themselves to the selection pressure exerted by BCAs in a few generations [203]. Biological control is frequently thought to have a longer lifespan than chemical control. This assumption may not always be justified, according to research on pest management in agricultural systems. Resistance of several pests to one or more Bt toxins, as well as the emergence of resistance of the codling moth *Cydia pomonella* to the *C. pomonella* granulovirus, have been documented.

In contrast to pest control, the persistence of biological control of plant diseases has received little attention, and no scientific papers demonstrating the loss of efficacy of BCAs against plant pathogens in practice have been reported yet. The success of BCAs against plant diseases may not be durable, as pathogen populations may develop resistance similar to that seen with single-mode chemical fungicides. Variation in susceptibility to the mode of action within the pathogen population, leading to changes in pathogen populations toward less sensitive strains due to selection pressure, and the fitness of the selected strains in the environment under conditions without selection pressure are all important factors in the deterioration of effectiveness [203]. For successful biocontrol, a weak link in the pathogen life cycle needs to be identified as an opportunity window. The antagonist should penetrate the opportunity window and disrupt the disease cycle. For some unspecialized necrotrophic pathogens, for example, the window of opportunity is to prevent the uptake of nutrients necessary for growth. When the antagonist competes for nutrients and prevents the saprophytic phase from establishing, this phase will be suppressed. The loss of endogenous nutrients from the pathogen spore may limit or prevent germination when antagonistic organisms are present in sufficient quantities in the area surrounding the pathogen spores. The synthesis of enzymes or antibiotics by the antagonist may also be effective against these diseases [32].

### 5.3. Effect of the Biocontrol Agent on Biocontrol Activity

The ability of BCAs to adapt to local biotic and abiotic environmental conditions is the main reason behind their diminished efficiency in the field. Therefore, the spatial distribution pattern of BCAs in the rhizosphere should be studied to fully understand this phenomenon [69]. Additionally, more suitable native strains of BCAs should be collected and screened to achieve relevant biocontrol results [67]. The efficiency of BCAs is closely linked to their modes of action [204] and frequently involves tradeoffs with other natural qualities of the agents, such as specificity and environmental persistence [31]. The most commonly observed hurdles in biocontrol include the microbial strain intrinsic biological properties (such as its “ecological competence”), as well as the quality of the formulated preparations utilized in the field or inadequate application time or method [201]. When an ideal relationship prevails, the biocontrol agent will be more effective. Therefore, for successful biocontrols, the application of the antagonist at the right time is critical. When the antagonist is applied before the pathogen’s establishment, the biocontrol will accomplish its goals [32]. Furthermore, to achieve optimal disease control, it is critical to understand how BCAs work. Understanding the mode of action is also necessary for identifying potential risks to humans or the environment, as well as risks of resistance development against the BCA [35]. Fluorescent pseudomonads have many features that make them ideal BCAs. These characteristics include (i) efficient colonization of roots, tubers, hypocotyls, and other parts of the plant; (ii) the ability to use a variety of organic substrates commonly found in root and seed exudates; (iii) ease of cultivation in the laboratory, (iv) production of a variety of secondary metabolites; and (v) compatibility with commonly used pesticides and other biocontrol agents [28].

### 5.4. Effect of the Environment on Biocontrol Activity

Soil biological and environmental factors have a significant impact on the success of biocontrol programs. Subsequently, soil biology research should identify the numerous significant characteristics of various organisms, particularly in the plant rhizosphere, by recognizing their relative contribution to the biocontrol process. Similarly, ecological research should look into all biotic and abiotic elements that influence the BCAs as vital components of plant health [205]. Biological control of postharvest diseases (causing losses up to 55%) through BCAs is a potentially sustainable control method. However, in comparison to synthetic chemical pesticides, there is a significant lack of reliability in field conditions. The biocontrol activity of BCAs has been improved by combining BCAs application to nutrient additives, salts, edible coatings, or physical treatments, but with only limited success. Complex microbial populations inhabit the fruit surface, which is frequently resilient. It may be hard to build a BCA in such an environment. Integrating the role of microbial communities in the formation of a BCA which is compatible with other soil microbiome is a viable option for managing BCAs dependability in realistic conditions. A microbial BCA is a complex metabolic phenotype that can be broken down into many processes, all of which are maintained by a complex network function in the soil. Combining BCAs application with a suitable complex biocontrol mix that includes beneficial helper strains, essential macro, micronutrients, and biocontrol prebiotics may aid the establishment of BCAs in the epiphytic microbial network. At the same time, it could be able to attain biocontrol efficacy and consistency on par with synthetic chemical pesticides. In addition, based on the literature, the time of beneficial microbial application has been examined [206]. Sare et al. [206] proposed, for example, that shifting treatment at the blooming stage (to establish a “path dependency”) be investigated for future fruit and vegetable postharvest disease management. Other plant organs, such as seeds, could benefit from this application moment shift [206]. In many cases, although plant-parasitic nematodes (PPNs) had demonstrated potential susceptibility in laboratory or field plots, satisfactory success was not attained [207]. Econem, a bionematicide containing in vitro produced *Pasteuria* sp., was found to be ineffective in the management of a PPN, *Belonolaimus longicaudatus* Rau, on golf course grass. In one of eight field experiments, the product only reduced *B. longicaudatus* populations on a single sample [208]. Timper et al. [202] highlighted examples of how agricultural practices can help or hinder the biological control of PPNs and other soil-borne pests. The effectiveness of nematode antagonists can be improved by supplying additional food sources, such as when organic amendments are provided to the soil. Some organic additions, such as manures and plants that contain allelopathic substances, can, however, be harmful to nematode antagonists. Crop rotation, fallow periods, tillage, and pesticide sprays are all examples of production practices that can disrupt antagonistic organism populations directly. By diminishing antagonists’ primary nematode host, these measures can have an indirect effect [202]. BCAs are being more widely utilized to combat a variety of PPN pests, and they are a safer alternative to toxic chemical nematicides. Despite this, due to their lack of efficacy, uneven field performance, and/or negative economic circumstances, they have been confined to a limited sector of the pesticide industry. A holistic understanding of soil biological and ecological components can improve the efficacy and biocontrol success including improved sampling, a better understanding of BCAs interactions with soil biota and ecology, cost-effective BCAs use, genetic manipulation for better PPNs control, grower acceptance and awareness of BCAs techniques, and commercial application [205].

## 6. Conclusions

The available data suggest that the assumption that biological control is always more sustainable than chemical control is not always valid. However, concluding the existence of unique features related to the plant pathogen or the BCA that could explain the BCA’s lack of effectiveness in practice is insufficient. Briefly, to ensure BCAs’ long-term efficacy against target plant diseases, it is critical to understand how their efficacy may be compromised. This knowledge will lead to the identification of risk factors that will promote the selection of plant pathogen strains that are resistant to BCAs. It will also lead to the identification of BCAs with a lower threat of failure efficacy, i.e., BCAs with modes of action that do not favor the selection of resistant isolates in natural plant pathogen populations [203]. Manipulation of the environment, the use of mixtures of beneficial organisms, physiological and genetic enhancement of the biocontrol mechanisms, formulation manipulation, and the integration of biocontrol with other alternative methods that provide additive effects can all help to improve the efficiency of these biocontrol products. These BCAs could be used effectively in sustainable agriculture to boost plant growth [28]. To ensure effective biocontrol, it is critical to choose agents that are effective in a variety of situations, including soil texture, wetness, temperature extremes, and competition [31]. In addition to the aforementioned factors, the need for scientists to publish negative data, as well as their ability to do so, appears to be critical to the success of biological control. The researchers will be able to determine biopesticides weaknesses such as lack of efficacy, uneven field performance, and/or unfavorable economic considerations to tackling them in the future [209].

## Data Availability

The data used for the analyses in this study are available within the article.
